# Untargeted serum metabolomics and tryptophan metabolism profiling in type 2 diabetic patients with diabetic glomerulopathy

**DOI:** 10.1080/0886022X.2021.1937219

**Published:** 2021-06-22

**Authors:** Fanliang Zhang, Ruixue Guo, Wen Cui, Li Wang, Jing Xiao, Jin Shang, Zhanzheng Zhao

**Affiliations:** a Department of Nephrology, the First Affiliated Hospital of Zhengzhou University, Zhengzhou, Henan, P.R. China; b Biobank of, The First Affiliated Hospital of Zhengzhou University, Zhengzhou, Henan, P.R. China

**Keywords:** Diabetic glomerulopathy, serum metabolites, mechanism, untargeted metabolomics

## Abstract

Diabetic glomerulopathy (DG) remains the prevalent microvascular complication and leading cause of shortened lifespan in type-2 diabetes mellitus (T2DM) despite improvement in hyperglycemia control. Considering the pivotal role of kidney in metabolism, using untargeted metabolomic techniques to globally delineate the serum metabolite profiles will help advance understanding pathogenetic underpinnings of renal biopsy-confirmed DG from the perspective of metabolism specifically. Fourteen pathologically diagnosed DG patients secondary to T2DM and 14 age- and gender-matched healthy controls (HCs) were recruited for study. We employed mass spectrometry-based untargeted metabolomic methods to reveal the metabolite profiles of serum samples collected from all included subjects. We identified a total of 334 and 397 metabolites in positive and negative ion mode respectively. One hundred and eighty-two important differential metabolites whose variable importance in projection (VIP) > 1 and *p* value <0.05 were selected and annotated to metabolic pathways. KEGG pathway enrichment analysis revealed *tryptophan metabolism* enriched most significantly. Among the tryptophan derivatives, L-tryptophan (L-Trp) and serotonin were relatively accumulated in DGs compared with HCs, while 5-hydroxyindoleacetic acid (5-HIAA) and indole-3-acetamide were depleted. Correlation analysis showed serotonin and L-Trp are negatively correlated with 24 h urine protein and glycosylated hemoglobin (Ghb). To exclude the interference of preexisting T2DM on DG exacerbation, we selected 5-HIAA and 3-(3-hydroxyphenyl) propionic acid (3-OHPPA) which are not correlated with Ghb and analyzed their correlation relationship with crucial renal indices. We found 3-OHPPA is positively correlated with urine total protein and creatinine ratio (T/Cr) and 24 h urine protein, 5-HIAA is positively correlated with serum creatinine and urea.

## Introduction

Diabetic nephropathy (DN) and its most devastating manifestation, end-stage renal disease (ESRD), remain one of the most common causes of shortened lifespan in diabetes mellitus (DM) population [1]. Diabetic glomerulopathy (DG), the pathognomonic lesions of DN, is defined as constellation of glomerular basement membrane (GBM) thickening, mesangial expansion and nodules formation, and afferent and efferent glomerular arteriolar hyalinosis [2]. The incidence of ESRD due to DG has alarmingly elevated over the past two decades despite improvement in hyperglycemia and hypertension control [3]. Thus, it is urgently needed to advance the understanding of DG pathogenesis.

Nowadays, emerging studies have corroborated the pivotal role of kidney in metabolism by regulating circulating metabolites secretion or reabsorption [4–6]. It has been reported that serum metabolomes of patients with ESRD caused by DN is different with healthy individuals [7]. Considering the wider spectrum of underlying possible glomerular lesions of the DN patients secondary to T2DM [2], it is necessary to delineate the serum metabolite profiles of renal biopsy-confirmed DG, which may aid in uncovering the pathophysiology of DG accurately.

Metabolomics, also known as metabonomics, is defined as the ‘quantitative measurement of the dynamic multi-parametric metabolic responses of living systems to pathophysiological stimuli or genetic modifications’ [8]. Untargeted metabolomic techniques can globally examine and quantify small molecules from various biological samples (e.g., urine, serum, feces, vitreous fluid, etc.) and provides the relatively unbiased approach to identify early biochemical alterations, unravel potential biomarkers and underlying pathogenetic mechanisms of multiple diseases, like chronic kidney disease, nephrotoxicity-induced acute kidney injury, renal cell carcinoma, autosomal dominant polycystic kidney disease and other ciliopathies [9–12]. To our knowledge, there is still no study about serum metabolomics analysis from pathologically diagnosed DG patients.

Ultraperformance liquid chromatography (UPLC) technology has been considered to be suitable for proteomics and metabolomics (like lipidomics) detection, particularly for universally untargeted metabolomics due to its high sensitivity in detecting metabolites [13,14]. Moreover, MS-based metabolomics combined with liquid chromatography (LC) can separate individual metabolite, which permits to detect low-concentration metabolites as well as identify them accurately [9]. During recent studies, UPLC-MS metabolomics technology has been widely applied to reveal the potential biomarkers in clinical chemistry, like renal, cardiovascular, and neuropsychiatric diseases and cancer [14–16].

The aim of our study is to delineate the serum metabolite profiles of DG patients using liquid chromatography-mass spectrometry-based untargeted metabolomic analysis technologies, uncover the relationship between metabolites and renal indices, and elaborate the role of significantly changed metabolites in DG pathogenesis.

## Methods and materials

### Ethical approval

All subjects included in this study signed informed consent. All procedures performed in studies involving human participants were in accordance with the ethical standards of the institutional and/or national research committee at which the studies were conducted. The Ethics Review Committee of First Affiliated Hospital of Zhengzhou University granted ethical approval for the research (2019-KY-361).

### Study design, participants recruitment and samples collection

**Figure 1.**
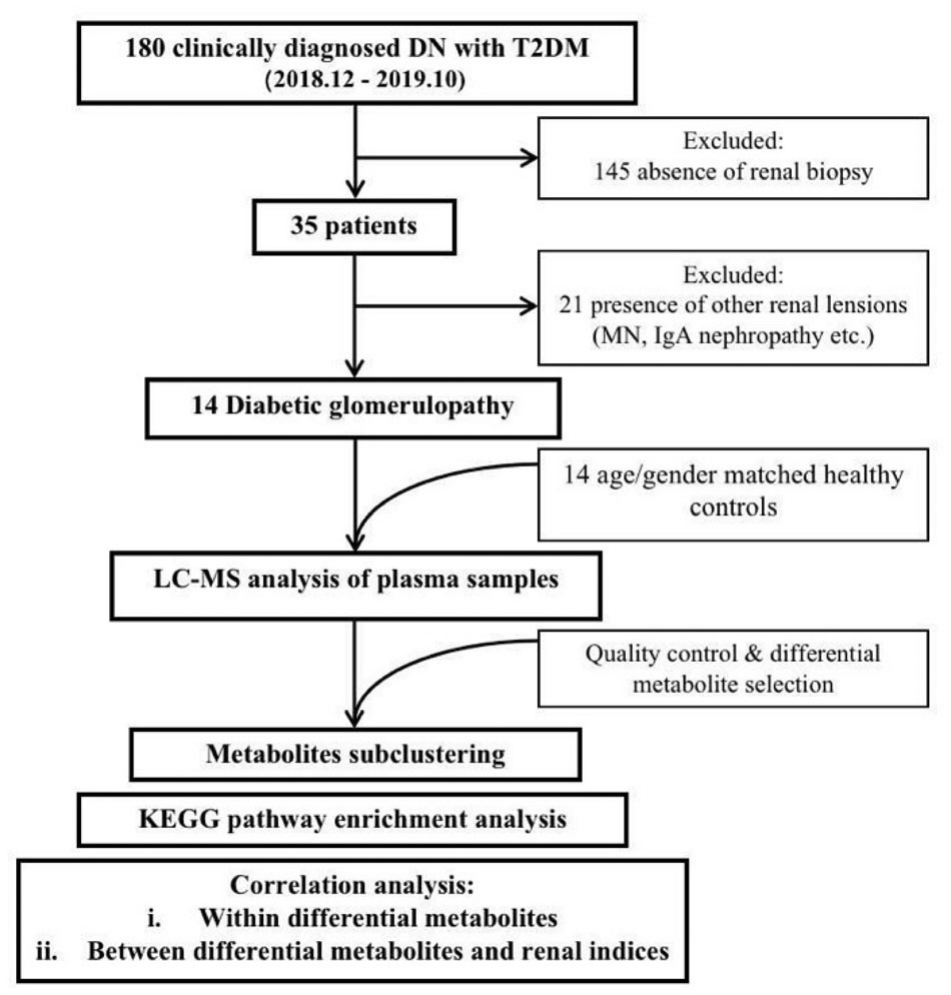
Flow chart of study design.

As shown in Figure 1, a total of 180 patients (18–75 years old) who were admitted in the First Affiliated Hospital of Zhengzhou University due to clinically diagnosed DN secondary to T2DM between December 2018 and October 2019 signed informed consent and were enrolled. The diagnostic criteria of DN were based on the American Diabetes Association guidelines and set as (1) estimated GFR (eGFR) <60 mL/min/1.73 m^2^ or (2) albuminuria >30 mg/g creatinine more than 3 months [17].

Thirty-five of the 180 enrolled subjects underwent renal biopsy and the results were evaluated by two pathologists, while rest of 145 were excluded. Then, 14 patients were pathologically diagnosed as DG and finally entered study cohort, while 21 patients who were confirmed as the renal lesions of other diseases (e.g., membranous nephropathy, IgA nephropathy, etc.) were excluded. Meanwhile, 14 age- and gender-matched healthy controls (HC) were recruited from healthy people sample center of the First Affiliated Hospital of Zhengzhou University.

The serum samples of both DGs and HCs were collected, temporarily conserved in ice packs and immediately sent to the Biobank of The First Affiliated Hospital of Zhengzhou University for cold storage at −80 ˚C environment within 2 h for further untargeted metabolomic analysis.

### The chemicals and equipment

The methanol, acetonitrile, and water were all obtained from Fisher Chemical (Shanghai, China). Formic acid was purchased from CNW. 2-Propanol was obtained from Merck. 2-Chloro-L-Pheylalanine was from Adamas-beta.

The centrifuge 5424 R and 5430 R were both purchased from Eppendorf (Shanghai, China). UHPLC liquid chromatography system (Vanquish Horizon System) and mass spectrometer (Q-Exactive HF-X) were both purchased from Thermo Scientific (Shanghai, China).

### Sample processing and quality control/QC

The samples were pre-processed to remove proteins and impurities. We transferred 100 µL sample into a 1.5-mL centrifuge tube, added 400 µL methanol containing 0.02 mg/mL internal standard (L-2-chlorophenylalanine). After vortexing and mixing for 30 s, the samples underwent low-temperature ultrasonic extraction (5 °C, 40KHz) and were frozen at −20 °C for half an hour, respectively. Then centrifuged samples for 15 min (13,000 g, 4 °C), supernatants were collected and transferred into injection sample vials for further computer analysis. During the test, the QC samples were evenly and randomly distributed in the injection process.

### UPLC-MS analysis

A Vanquish Horizon ultra-high-performance liquid chromatography system (Thermo Scientific) was equipped with ACQUITY UPLC HSS T3 column (100 mm × 2.1 mm i.d., 1.8 µm; Waters, Milford, USA). The binary gradient elation system consisted of mobile phase (A), which is composed of 95% water and 5% acetonitrile (containing 0.1% Formic acid), and (B), which is composed of 47.5% acetonitrile, 47.5% isopropanol and 5% water (containing 0.1% Formic acid). The separation was achieved using following gradient: 0–100% B over 0–5.5 min, the composition was held at 100% B at 5.5–7.4 min, then 7.4–7.8 min, 100% to 0 B, and 7.8–10 min holding at 0 B. The flow rate was 0.4 mL/min, and the column temperature was 40 °C, the injection volume was 2 µL.

Mass spectrometry was performed on a Q-Exactive HF-X system (Thermo Scientific). The mass range was from m/z 70 to 1050. The resolution was set at 60 000 for the full MS scans and 7500 for MS^2^ scans. The samples were ionized by electrospray and the mass spectrometry operated as follows: spray voltage, 3500 V (positive) and 3500 V (negative); sheath gas flow rate, 50 arbitrary units; auxiliary gas flow rate, 13 arbitrary units; capillary temperature, 325 °C.

### Bioinformatic and statistical analysis

The original metabolomic data were processed using the Progenesis QI (WaterCorporation, Milford, USA), which produced a matrix features with retention time, peak area, mass-to-charge ratio and identification information. All variables were normalized to the total peak area of each sample. All metabolites were identified by MS and MS/MS fragment through Progenesis QI (WaterCorporation, Milford, USA) with several mainstream public databases (http://www.hmdb.ca/, https://metlin.scripps.edu/). Afterwards, the ProgenesisQI (WaterCorporation, Milford, USA) was used to search and identify the characteristic peaks. We set MS mass error as less than 10 ppm, matched the MS and MS/MS mass spectrum information with the metabolic database, then the metabolites were identified based on the secondary mass spectrometry matching score. The main databases used for metabolites identification are several mainstream public databases (http://www.hmdb.ca/, https://metlin.scripps.edu/). Principle component analysis (PCA) and Orthogonal Partial Least-Squares Discrimination Analysis (OPLS-DA) were performed to identify the discrimination of variables. Permutation testing was used to evaluate the accuracy of PLS/OPLS-DA. Based on OPLS-DA analysis, the metabolites with variable importance in projection (VIP) >1 are recognized as important variables. VIP represents the ability to extract variables of differentiation between DG and HC groups. Important differential metabolites were defined as those with VIP >1.0 obtained from OPLS-DA and adjusted *p* values <0.05. Hierarchical cluster analysis (HCA) was applied to create heatmaps of the differentially expressed metabolites and to assign metabolites to clusters (R version, package gplot), combined with Spearman and Pearson correlation analyses. Based on HMDB, KEGG and LIPID MAPS databases, all important differential metabolites were annotated to specific pathways and classified based on pathways’ function. KEGG pathway topology was applied to evaluate the extent of important differential metabolites’ influence on their functional pathways.

Data collected from biochemical assay were expressed as mean ± SEM. Statistical analyses were performed using SPSS 23.0 software. Comparisons between groups were measured by Student’s *t*-test. *p* Values <0.05 were considered statistically significant.

## Results

### Baseline characteristics of recruited DGs and HCs

As mentioned in *Methods*, 14 pathologically diagnosed DGs and 14 age- and gender-matched HCs were recruited. As shown in Table 1, we recruited the DGs whose fasting blood sugar level were comparable with HCs’, which is due to blood glucose was well controlled around sample collection. As for medication history, 3 of 11 DGs received regular insulin therapy, four of 10 DGs used oral antidiabetic drugs (OADs, one used metformin, one used dipeptidyl peptidase (DPP)-4 inhibitors, one used meglitinides, one used alpha-glycosidase inhibitors and DPP-4 inhibitors), one of 13 received antibiotics. Even though significant difference of OADs application between DGs and HCs exists, it has been reported that OADs do not impact serum metabolomics of DN patients significantly [7]. Thus, we excluded the interference of medications perfectly. The laboratory indices (including glycosylated hemoglobin, serum creatinine and urea, 24-h urine protein and urine total protein and creatinine ratio) of DGs are significantly higher than HCs. The blood lipid profiles were totally matched.

**Table 1.** Participants demographic and clinical features.

	DN (*n* = 14)	HC (*n* = 14)	*p* Value
Age(years)	50.29 ± 10.29	48.93 ± 10.49	0.733
Gender(male/female)	8/6	7/7	0.314
oral hypoglycemic drugs(yes/no)	4/10	0/14	0.031
Insulin (yes/no)	3/11	0/14	<0.01
Antibiotic use (yes/no)	1/13	0/14	0.309
Ghb(%)	7.50(6.76,8.59)	5.85(5.75,5.90)	<0.01
Hb(g/L)	105.58 ± 19.01	136.71 ± 13.99	<0.01
Count of NG (10^9/L)	3.55 ± 1.24	3.26 ± 0.77	0.468
Count of Leu (10^9/L)	1.52 ± 0.70	1.95 ± 0.32	0.056
24h-Protein (g/24h)	5.64(2.82,8.25)	0(0,0)	<0.01
TCR（g/g)	3.84(2.03,8.92)	0(0,0)	<0.01
Blood glucose (mmol/L)	5.53(4.41,7.96)	4.98(4.82,5.11)	0.227
Urea (mmol/L)	9.66(6.52,13.07)	3.98(3.68,4.98)	<0.01
Creatinine (μmol/L)	226.64 ± 318.63	72.57 ± 20.92	0.094
Uric acid (μmol/L)	320.14 ± 70.63	281 ± 97.57	0.235
eGFR (mL/min/1.73 m^2)	56.07 ± 29.83	95.45 ± 12.48	<0.01
Alb (g/L)	30.78 ± 6.36	46.5 ± 2.87	<0.01
CHO (mmol/L)	4.6 ± 1.72	4.44 ± 0.84	0.762
TG (mmol/L)	1.55(1.05,2.34)	1.04(0.62,1.51)	0.164
HDL (mmol/L)	1.4 ± 0.33	1.15 ± 0.48	0.123
LDL (mmol/L)	3.06(1.56,3.82)	2.84(1.98,3.24)	0.635
DM course (months)	120(99,180)	0(0,0)	<0.01
Hypertension history (months)	18(1.62,66)	0(0,0)	<0.01
CD history (months)	0(0.27)	0(0,0)	0.21
CI course (months)	0(0,0)	0(0,0)	0.769
DR (positive/negative)	0/6	/	/

Ghb: glycosylated hemoglobin; Hb: hemoglobin; NG: neutrophilic granulocyte; Leu: leukocyte; TCR: urine total protein and creatinine ratio; eGFR: estimated glomerular filtration rate; Alb: serum albumin; CHO: cholesterol; TG: triglyceride; HDL: high-density lipoprotein; LDL: low-density lipoprotein; DM: diabetes mellitus; CD: cardiovascular disease; CI: cerebral infarction; DR: diabetic retinopathy.

### Disparity of serum metabolomics between DGs and HCs

**Figure 2.**
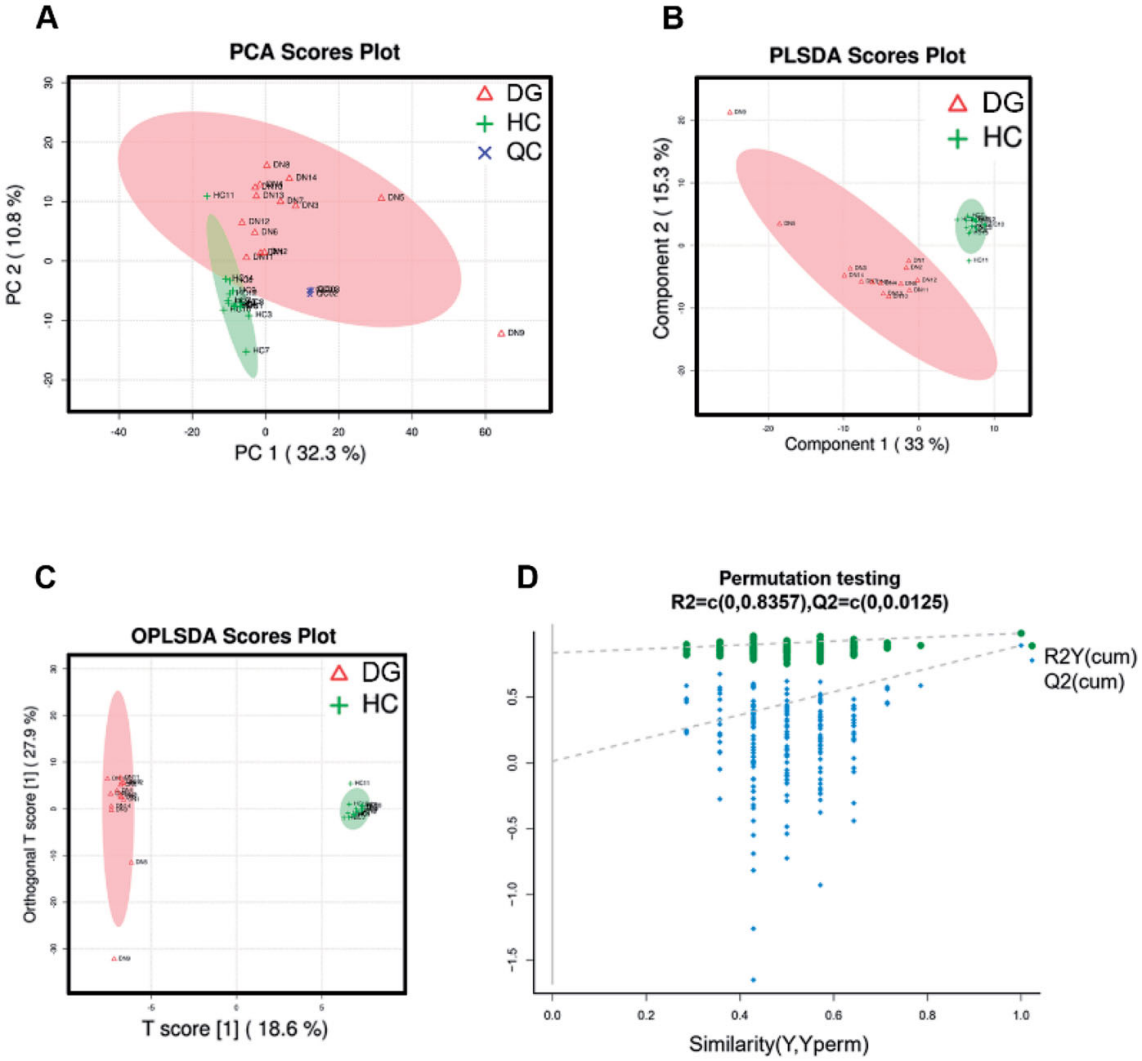
Analysis and validation of serum metabolites disparity between DGs and HCs. (A) PCA scores plots; (B) PLS-DA scores plot; (C) OPLSA-DA scores plot; (D) Scatter plots of the statistical validations obtained by 200’s permutation tests.

We employed UPLC-MS on DGs’ and HCs’ serum samples and identified a total of 334 and 397 metabolites in positive and negative ion mode respectively. Serum metabolic characteristics were first evaluated using multivariate statistical analyses. The stability of samples collection and handling were evaluated by internal QC. PCA provided an unsupervised and comprehensive view of serum samples. Significant disparities were illustrated between DG and HC groups, with an acceptable explanatory value of PCA model (accumulative *R*^2^*X* = 0.51, Figure 2(A)). Furthermore, PLS-DA analysis was performed to maximally analyze the difference and confirmed marked altered serum metabolite profiles between DG and HC groups (*R*^2^*Y* = 0.985, *Q*^2^ = 0.892, Figure 2(B)). Finally, we used OPLS-DA to maximize the class discrimination and calculate the VIP value of each metabolites. OPLS-DA showed a complete separation (*R*^2^*X* = 0.55, *R*^2^*Y* = 0.997, Figure 2(C)) once again. In 200’s permutation test, all *R*^2^ and *Q*^2^ values of permutated models were worse than original model, indicating a better prediction ability and reliability of this model (Figure 2(D)). Therefore, we revealed the serum metabolite profiles of DG patients has dramatically altered compared with HCs.

### Serum metabolite profiles identification

**Figure 3.**
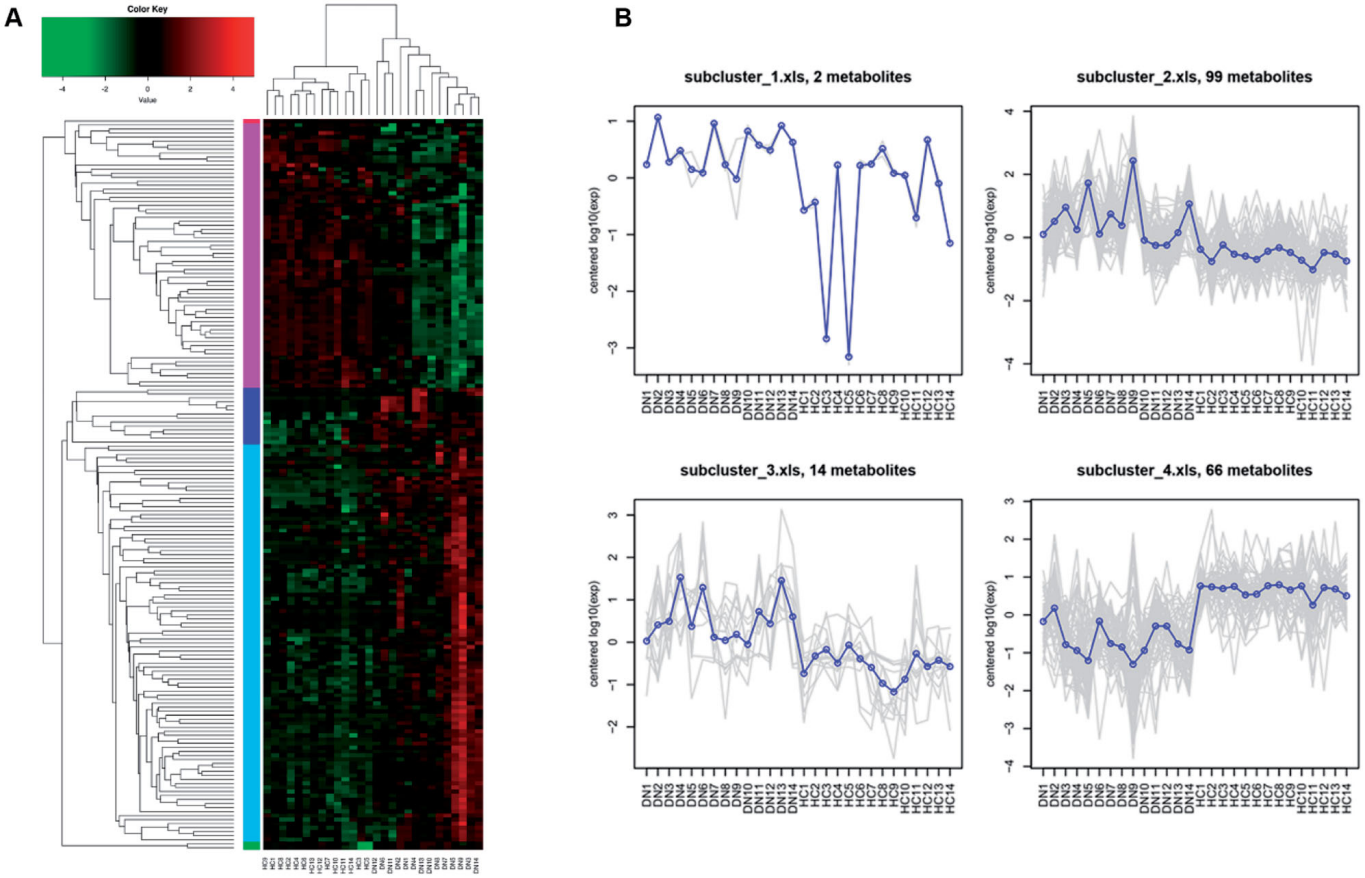
HCA for important differential metabolites between DGs and HCs. (A) Hierarchical clustering and heatmap of all 182 metabolites that were identified to be significantly different (*p* ≤ 0.05) in concentration between DGs (*n* = 14) and HCs (*n* = 14). (B) Trend line charts of 4 subclusters presenting average expression level of the important differential metabolites they contained.

To select the metabolites as potential candidates which may participate in DG pathophysiology and influence renal indices, we combined OPLS-DA analysis with Student’s *t*-test. We screened in the 182 metabolites whose VIP are greater than 1 and *p* value are less than 0.05 and defined them as important differential metabolites. Among them, there are 87 metabolites in ESI + mode and 95 in ESI- mode. The relative expression level of 182 metabolites from each sample were presented as heat map (Figure 3(A)). Based on expression mode, these metabolites could be further divided into five clusters, and the average expression level of them were shown in Figure 3(B). We found subclusters 2 and 3 (containing 99 and 14 types of metabolites, respectively) were obviously enriched in DG patients’ serum sample, while subcluster 4 (containing 66 types of metabolites) was relatively depleted compared with HCs.

### Enriched metabolic pathways analysis

**Figure 4.**
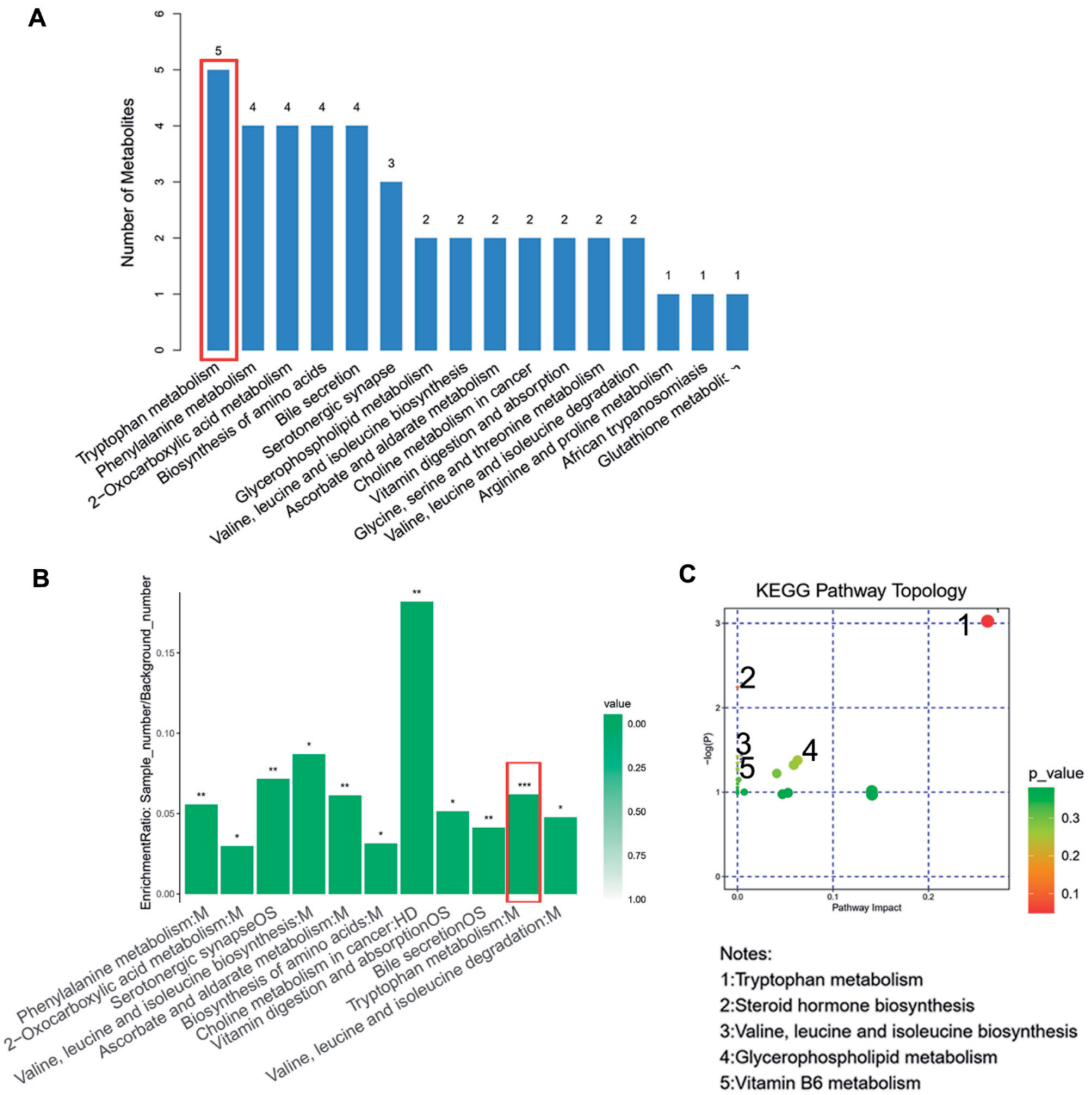
Advanced metabolic pathways analysis. (A) The top 16 metabolic pathways ranked by the number of important differential metabolites they contained. (B) The KEGG enrichment analysis of all 11 significantly enriched metabolic pathways. (C) The bubble chart of KEGG pathway topology indicating the tryptophan metabolism are influenced most notably by its important differential metabolites.

**Figure 5.**
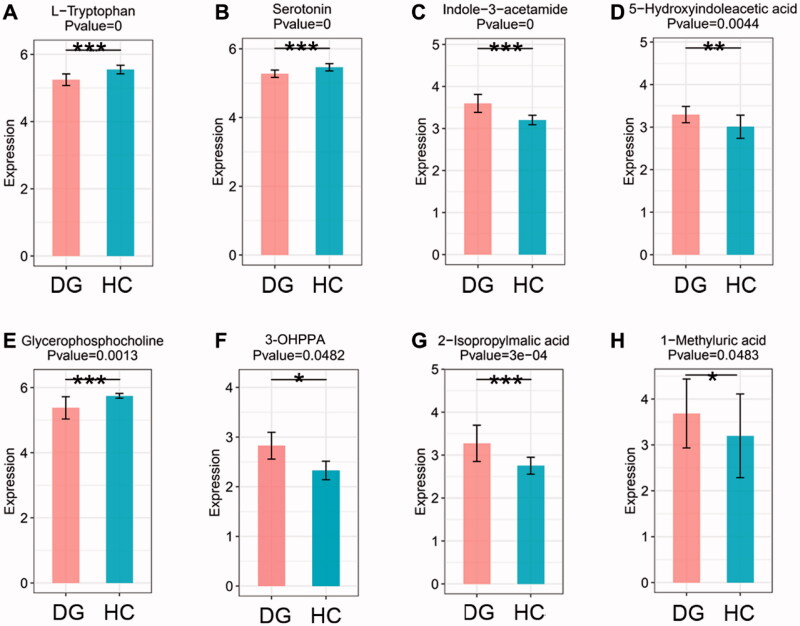
The boxplots showing the comparison of the relative expression level of four important differential metabolites of tryptophan metabolism and other four selected metabolites between DGs and HCs. (A) L-Tryptophan. (B) Serotonin. (C) Indole-3-acetamide. (D) 5-Hydroxyindoleacetic acid. (E) Glycerophopshocholine. (F) 3-(3-hydroxyphenyl)propionic acid (3-OHPPA). (G) 2-Isopropylmalic acid. (H) 1-methyluric acid. *p < 0.05, **p < 0.01 and ***p < 0.001 vs. HCs. M: metabolism; HD: human diseases; OS: organismal system.

By searching KEGG/HMDB database, we obtained the pathways in which 182 important differential metabolites are involved. Among the top 16 enriched metabolic pathways ranked by the number of important differential metabolites they contained, *tryptophan metabolism* contained the largest number of metabolites, which are five types of metabolites, followed by *phenylalanine metabolism* and *Biosynthesis of amino acids* (Figure 4(A)). KEGG pathway enrichment analysis further confirmed the results above and revealed that enrichment of *tryptophan metabolism* is most significant (*p* < 0.001, Figure 4(B)). We evaluated the degree of important differential metabolites’ influence on their pathway *via* MetPA analysis. The results showed that tryptophan derivatives have the most notable impact on *tryptophan metabolism* (Figure 4(C)). The expression level of four important differential metabolites belonging to *tryptophan metabolism* were compared between DGs and HCs. L-tryptophan (L-Trp) and serotonin were relatively depleted in DGs compared with HCs (Figure 5(A,B)), while indole-3-acetamide and 5-hydroxyindoleacetic acid (5-HIAA) were relatively accumulated (Figure 5(C,D)). It is noted that 3-(3-hydroxyphenyl) propionic acid (3-OHPPA), the metabolite belonging to *phenylalanine metabolism*, is relatively enriched in DGs than HCs (Figure 5(F)). Of the remaining important differential metabolites, we found glycerophosphocholine (GPC) was relatively decreased in DGs than HCs (Figure 5(E)), while 2-isopropylmalic acid and 1-methyluric acid were relatively increased (Figure 5(G,H)).

### Correlation analysis

**Figure 6.**
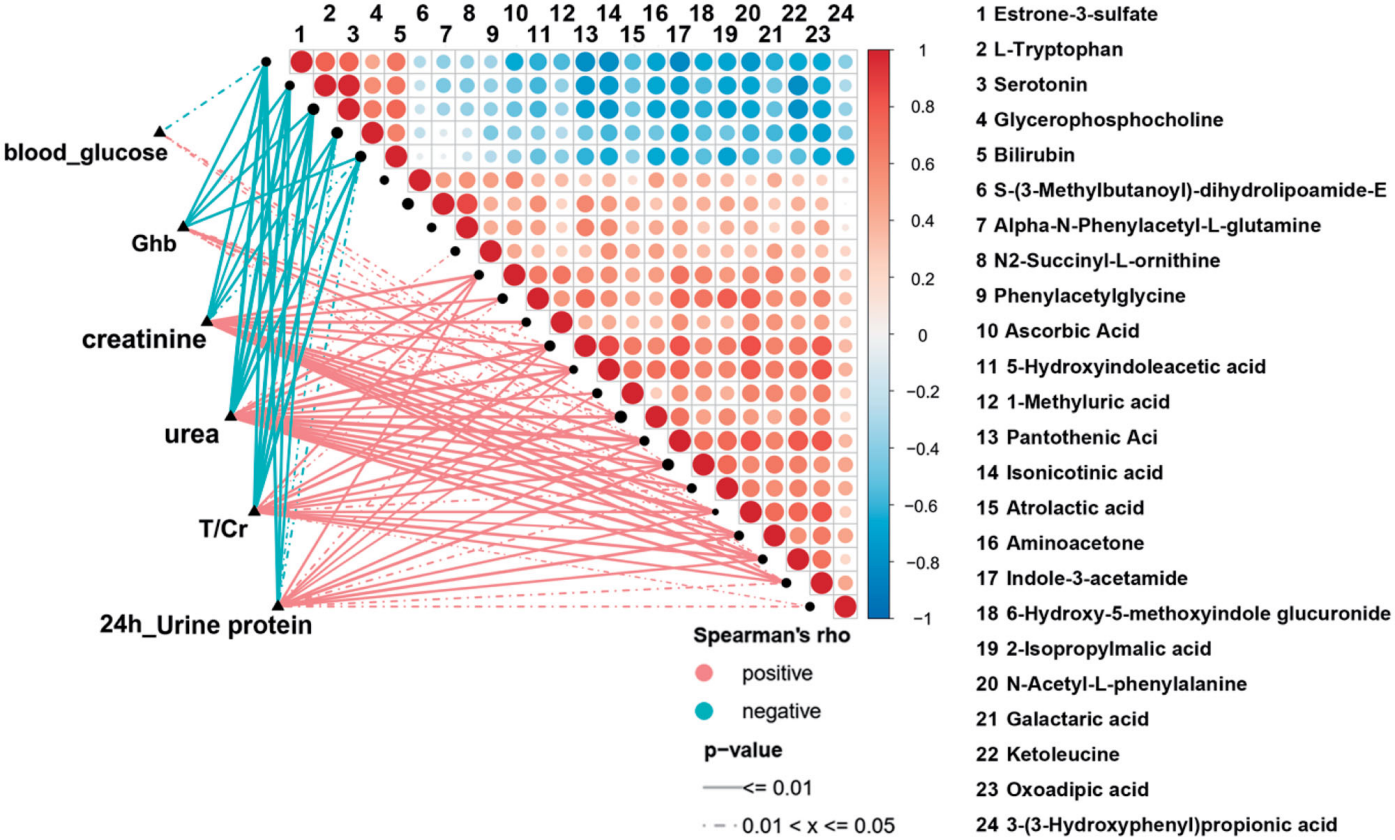
The heatmap of the correlation analysis between 24 selected important differential metabolites (containing 4 important differential metabolites of tryptophan metabolism) and 6 crucial renal indices (i.e., blood glucose, urea, Ghb, T/Cr and 24 h urine protein). Ghb: glycosylated hemoglobin; T/Cr: urine total protein and creatinine ratio.

**Figure 7.**
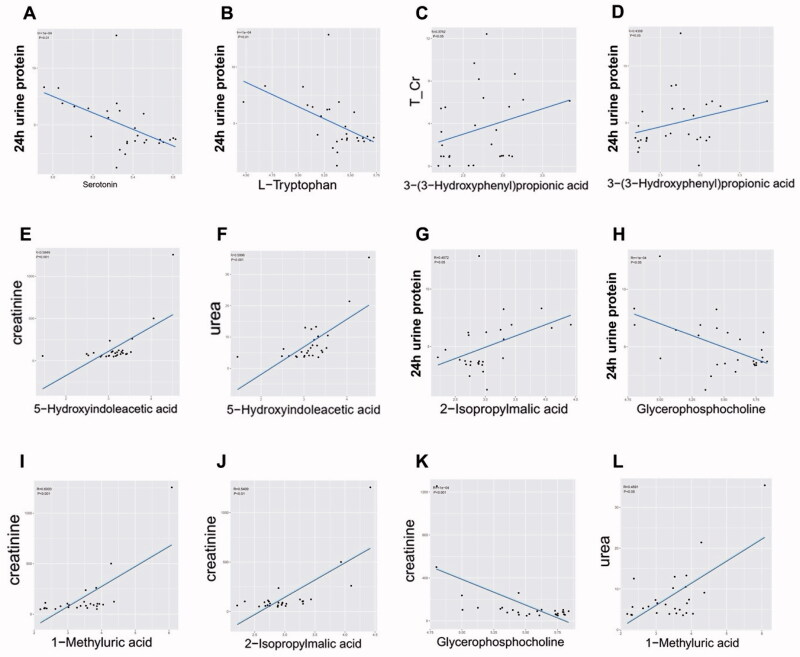
A-L. The linear regression plots of the correlation relationship between 7 selected important differential metabolites (containing 3 important differential metabolites of tryptophan metabolism) and 4 crucial renal indices (i.e., 24 h urine protein, T/Cr: serum urea and creatinine). T/Cr: urine total protein and creatinine ratio.

To illustrate the relationship within important differential metabolites and unravel their interaction with some crucial renal indices, we implemented Spearman correlation analysis. We analyzed the correlation relationship between 24 selected important differential metabolites (i.e., the derivatives of *tryptophan* and *phenylalanine metabolism* and other selected metabolic pathways) and DG-related clinical indices (Figure 6). Among tryptophan derivatives, we found serotonin and L-Trp are negatively correlated with 24 h urine protein (*p* < 0.01, rho = −0.568; *p* < 0.01, rho = −0.563, respectively. Figure 7(A,B)) but also with glycosylated hemoglobin (Ghb) (Figure 6). While 5-HIAA is positively correlated with serum creatinine and urea (*p* < 0.01, rho = 0.595; *p* < 0.01, rho = 0.600, respectively. Figure 7(E,F)) and not correlated with Ghb (Figure 6). As for *phenylalanine metabolism*, 3-OHPPA showed a clear positive correlation with urine total protein and creatinine ratio (T/Cr) and 24 h urine protein (*p* < 0.05, rho = 0.374; *p* < 0.05, rho = 0.431 respectively. Figure 7(C,D)), and it neither correlated with Ghb (Figure 6). Then, we analyzed the correlation relationship within the selected metabolites above. It is noted that serum 5-HIAA level is negatively correlated with L-Trp and serotonin (*p* < 0.01, rho = −0.572; *p* < 0.01, rho = −0.574, respectively Figure 6).

Beyond tryptophan and phenylalanine metabolism, we also selected 4 important differential metabolites belonging to other metabolic pathways based on the same criteria above. Among them, GPC was negatively correlated with 24 h urine protein and serum creatinine (*p* < 0.05, rho = −0.397; *p* < 0.001, rho = −0.590, respectively. Figure 7(H,K)), 2-isopropylmalic acid was positively correlated with 24 h urine protein and serum creatinine (*p* < 0.05, rho = 0.407; *p* < 0.01, rho = 0.541, respectively. Figure 7(G,J)), and 1-methyluric acid was positively correlated with serum creatinine and urea (*p* < 0.01, rho = 0.600; *p* < 0.05, rho = 0.459, respectively Figure 7(I,L)).

## Discussion

Even though hyperglycemia and hypertension control has been improved in DG management, the incidence of ESRD due to DG is still horribly increasing over the past two decades [3]. Considering the pivotal role of kidney in metabolism, our study revealed that significant serum metabolomic disparities exist between the pathologically diagnosed DG secondary to T2DM and general healthy individuals, and tryptophan derivatives may be served as pathogenic factors which worth being illustrated in depth.

By using LC-MS analysis, we globally delineated the serum metabolite profiles of DGs and uncovered that composition of serum metabolites alters significantly compared with HCs. PCA and PLS-DA analysis further confirmed the marked disparities, which is consistent with studies indicating robust changes of serum metabolites occurred in DN [7,18–23]. Impaired renal regulating effects of metabolism due to DM causes systemic metabolism disturbances, renal complications further alter serum metabolite profiles subsequently.

Based on OPLS-DA analysis combined with Student’s t-test, 182 important differential metabolites were selected and subclustered. Not surprisingly, different subclusters presented distinct expression level between DGs and HCs. In another word, the serum metabolomics of DG patients may change toward the ‘certain trend’. Therefore, we speculated reasonably that this ‘certain trend’ of alteration may participate in DG pathogenesis.

To illustrate this, we analyzed the enriched metabolic pathways containing important differential metabolites by searching KEGG/HMDB database. Marked altered metabolic function existed in DGs. It is noted that *tryptophan metabolism*, the most significantly changed pathway containing largest number of important differential metabolites (five types), is the most important metabolic pathway according to MetPA analysis. When compared with HCs, the level of L-Trp and serotonin are relatively lower, while 5-HIAA and indole-3-acetamide are higher.

From the clinical aspect, Spearman correlation analysis revealed several important differential metabolites are associated with the severity of DG manifestations. Serum level of serotonin and L-Trp are negatively correlated with 24 h urine protein. Since they are also correlated with Ghb, it is difficult to clarify whether preexisting DM state or DG is the major insult aggravating renal manifestations. Considering studies have revealed altered serum level of tryptophan and serotonin are ubiquitous in DM and its other microvascular complications (e.g., diabetic retinopathy, etc.) [24–28], it is reasonable to speculate that altered serum level of them may be not the solely cause of renal manifestations exacerbation in DG.

Therefore, we selected the metabolites which are not correlating with Ghb but only with crucial DG-related renal indices. The results showed 5-HIAA is only positively correlated with serum creatinine and urea (the indices indicating renal function), and 3-OHPPA is only positively correlated with 24 h urine protein. After excluding the interfering effects of DM, these renal indices-related metabolites may be served as candidates to provide new insights into studies on DG pathogenesis. 5-HIAA acts as vasoconstrictor potentially exacerbating microvascular injury and has been recognized as valuable biomarkers for estimating the DN-related risk during the early stages of the disease [27–30]. It is conceivable that relatively accumulated serum 5-HIAA level will worsen renal function in DGs characterized as elevating serum creatinine and urea level. However, 3-OHPPA, a gut microbiota-derived “protective” potent vasodilator [31], are relatively accumulated in DGs’ serum and positively correlated to 24 h urine protein based on this study. At the same time, studies reported that composition and metabolic function of DN patients’ gut microbiota alters significantly [7]. Thus, it is plausible to infer that more severe manifestation of DG (e.g., higher level of 24 h urine protein, etc.) may result in more dramatically enhanced 3-OHPPA production from gut microbiota to overcome DG deterioration and ‘protect’ host. Further deciphering the biological effects of these molecules will not only aid in understanding DG pathogenesis from the perspective of metabolism specifically, but also guide to concentrate on the effects of gut microbiota on DG and host metabolism.

Notably, the derivatives of *tryptophan metabolism* are closely associated. Pathological DG state will change the trend of inter-conversion among these metabolites. Besides used for protein translation, L-Trp is also catabolized to crucial secondary metabolites *via* two parallel pathways, which can be termed by their respective end-products or intermediates, that is, the serotonin and kynureine pathway [32]. According to previous studies, the serum level of both L-Trp and serotonin are elevated in DM and DN state [25–29]. Intriguingly, our study revealed decreased serum L-Trp and serotonin in DGs than HCs. Therefore, it may be the best time to inspiringly illustrate the unique mode of *tryptophan metabolism* in renal biopsy-confirmed DGs, rather than clinically diagnosed DNs.

5-HIAA, the end-product of serotonin pathway, has been confirmed relatively accumulated in DGs’ serum in this study. Spearman correlation analysis showed clear negative correlation relationship exists between the serum level of serotonin and 5-HIAA. Thus, it is conceivable to conclude that reduced serotonin level results from its enhanced degradation into 5-HIAA in DG state. Serotonin, which can be catabolized by kidney into weaker vasoconstrictor 5-HIAA [30,33], has been also reported involved in the pathological process of platelet aggregation [34] and thrombogenesis [35] in DM-induced vascular complications. Thus, the unique mode of enhanced degradation of serotonin into 5-HIAA in pathologically diagnosed DG may act as the self-protective process trying to overcome the metabolic disturbance and alleviating renal manifestations.

The pivotal role of tryptophan metabolism and its intermediates in the pathogenesis of kidney disease have been illustrated in many previous studies. Phenyl sulfate, the gut microbiota-derived intermediates of tryptophan, has been reported to contribute to albuminuria and podocyte damage in diabetic rat models. In a diabetic patient cohort, phenyl sulfate levels significantly correlate with basal and predicted 2-year progression of albuminuria in patients with microalbuminuria [36]. According to the study implemented by Zhao et al. [37], significantly altered tryptophan metabolism was also observed in chronic kidney disease rat models, indicating the crucial role of tryptophan metabolism in CKD. Moreover, the metabolites of tryptophan metabolism can act as the ligands binding to specific receptors to elicited various pathologic cellular process. For example, kynurenine, serotonin and indole derivatives may bind to aryl hydrocarbon receptor (AHR), a cytoplasmic ligand-activated transcription factor, to trigger oxidative stress, inflammation in chronic kidney disease [38]. *via* UPLC-based metabolomics technology, we constructed the important position of *tryptophan metabolism* once again and revealed novel metabolites derived from tryptophan in DG, which may provide fruitful insights into the study on DG pathogenesis.

Beyond the derivatives of *tryptophan* and *phenylalanine metabolism* mentioned above, we also selected other four types of significant differential metabolites of various metabolic pathways based on the same criteria. Broader metabolites belonging to different metabolic pathways were identified to influence renal function, which indicated that global metabolic imbalance are involved in DG pathogenesis.

GPC is the intermediates of choline metabolism which can be divided into 4 major pathways involving in the synthesis of phospholipids (e.g., phosphotidylcholine), trimethylamine (TMA), betaine and acetylcholine [39]. Our study revealed that the GPC level was decreased in DGs than HCs and negatively correlated with T/Cr, 24 h urine protein and serum creatinine, indicating that depleted GPC acts as exacerbating insult of DG renal manifestation. Considering GPC is the downstream product of phosphatidylcholine and TMA is converted by liver into trimethylamine-*N*-oxide (TMAO) which acts as uremic toxins and has been confirmed accumulated in DNs’ serum [40,41], we infer reasonably that decreased GPC level is caused by shunting of choline resource from synthetic pathway of phospholipids toward TMA. Meanwhile, enhanced TMA production will decrease the level of betaine and GPC simultaneously, which are the crucial methyl donor and major component of cell membrane respectively. Decreased level of them results in unstable cell structure and abnormal cell cycle [42–44], which may participate in DG pathogenesis and renal function exacerbation.

One-methyluric acid is the major physiological metabolite of caffeine [45] and annotated to *caffeine metabolism* according to our results. A case report described a heavy coffee consumer with high urine 1-methyluric acid level and suffered rare kidney stone composed of 1-methyluric acid [46]. As demonstrated in our study, 1-mthyluric acid was relatively accumulated in DGs than HCs and positively correlated with 24 h urine protein and serum creatinine. It may result from DG-induced *caffeine metabolism* imbalance. Meanwhile, elevated serum 1-methyluric acid may also injure endothelium and renal tubular cells, increase the risk of kidney stone, and aggravate renal function. Our study suggested caffeine restriction may be warranted for DG management.

The study on physiologic function of 2-isopropylmalic acid is limited. Intriguingly, it derives from metabolism of gut microbiota [47,48]. Our study unraveled the relatively increased serum level and correlation relationship with renal function exists in DG patients, suggesting the potential link between DG pathogenesis and gut microbiota once again.

The limitation of this study is relatively small number of subjects included in cohort as well as the limited number of serum samples. We will expand the size of cohort and use multivariate analysis to exclude the potential clinical indices indicating renal function which may influence metabolite profiles. Moreover, we have revealed the correlation relationship between the critical clinical renal indices of DG and important differential metabolites of *tryptophan metabolism*, indicating the critical role of *tryptophan metabolism*. Our following study will develop a larger DG cohort to validate the findings of this research once again and focus on the impact of tryptophan metabolic pathway on underlying pathogenetic mechanism of DG.

This study demonstrated the dramatically altered serum metabolite profiles of pathologically diagnosed DG using untargeted metabolomic techniques. Specifically, we constructed the critical position of *tryptophan metabolism* which may be served as the biomolecular candidates for future research on DG pathogenesis.
